# Latent Semantic Indexing of PubMed abstracts for identification of transcription factor candidates from microarray derived gene sets

**DOI:** 10.1186/1471-2105-12-S10-S19

**Published:** 2011-10-18

**Authors:** Sujoy Roy, Kevin Heinrich, Vinhthuy Phan, Michael W Berry, Ramin Homayouni

**Affiliations:** 1 Department of Computer Science, University of Memphis, Memphis, TN 38152, USA; 2 Bioinformatics Program, University of Memphis, Memphis, TN 38152, USA; 3 Department of Biology, University of Memphis, Memphis, TN 38152, USA; 4 Computable Genomix, Memphis, TN 38163, USA; 5 Department of Electrical Engineering and Computer Science, University of Tennessee, Knoxville, TN 37996, USA

## Abstract

**Background:**

Identification of transcription factors (TFs) responsible for modulation of differentially expressed genes is a key step in deducing gene regulatory pathways. Most current methods identify TFs by searching for presence of DNA binding motifs in the promoter regions of co-regulated genes. However, this strategy may not always be useful as presence of a motif does not necessarily imply a regulatory role. Conversely, motif presence may not be required for a TF to regulate a set of genes. Therefore, it is imperative to include functional (biochemical and molecular) associations, such as those found in the biomedical literature, into algorithms for identification of putative regulatory TFs that might be explicitly or implicitly linked to the genes under investigation.

**Results:**

In this study, we present a Latent Semantic Indexing (LSI) based text mining approach for identification and ranking of putative regulatory TFs from microarray derived differentially expressed genes (DEGs). Two LSI models were built using different term weighting schemes to devise pair-wise similarities between 21,027 mouse genes annotated in the Entrez Gene repository. Amongst these genes, 433 were designated TFs in the TRANSFAC database. The LSI derived TF-to-gene similarities were used to calculate TF literature enrichment p-values and rank the TFs for a given set of genes. We evaluated our approach using five different publicly available microarray datasets focusing on TFs *Rel*, *Stat6*, *Ddit3*, *Stat5* and *Nfic*. In addition, for each of the datasets, we constructed gold standard TFs known to be functionally relevant to the study in question. Receiver Operating Characteristics (ROC) curves showed that the log-entropy LSI model outperformed the *tf*-normal LSI model and a benchmark co-occurrence based method for four out of five datasets, as well as motif searching approaches, in identifying putative TFs.

**Conclusions:**

Our results suggest that our LSI based text mining approach can complement existing approaches used in systems biology research to decipher gene regulatory networks by providing putative lists of ranked TFs that might be explicitly or implicitly associated with sets of DEGs derived from microarray experiments. In addition, unlike motif searching approaches, LSI based approaches can reveal TFs that may indirectly regulate genes.

## Introduction

High throughput experimental approaches such as DNA microarray technology are expected to yield new discoveries. Gene expression profiling can identify hundreds of genes whose expression levels are co-regulated with experimental treatments. These experiments enable investigators to deduce functional pathways and regulatory mechanisms related to the observed genes and form the basis for new hypotheses that can be tested experimentally. A key step in this process is the identification of putative transcription factors (TFs) that are responsible for regulation of gene sets.

The vast majority of current methods focus on identification of DNA binding sites (motifs) of various TFs in the promoters of the co-expressed genes. For instance, Web-based tools such as CORE_TF [1] and oPOSSUM [2] identify overrepresented TF binding sites for gene sets. Experimentally derived consensus binding sites for many TFs can be obtained from commercial databases such as TRANSFAC [3] and Genomatix [4], or free ones such as JASPAR [5].

It is, however, important to note that presence of TF binding sites in gene promoters does not necessary imply a regulatory role. TF binding can depend on a number of other factors such as presence of competing TFs, and DNA structure [6,7]. Moreover, a TF may indirectly regulate a set of genes, for example, by binding to promoters of other TFs and inducing their expression, which in turn lead to regulation of the observed set of genes. It is, therefore, important to investigate alternative approaches to identify critical TFs from microarray data. While some of the differentially expressed genes (DEGs) and TFs may be known to functionally interact, it is expected that many interactions are implied, meaning the interaction is not verified experimentally and weakly supported in the literature. Therefore, there is a growing need to develop new text-mining tools to assist researchers in discovering hidden or implicit functional information about interaction of genes and TFs from the biomedical literature.

Information retrieval (IR) is a key component of text mining [8]. It consists of three types of models: set-theoretic (Boolean), probabilistic, and algebraic (vector space). Documents in each case are retrieved based on Boolean logic, probability of relevance to the query, and the degree of similarity to the query, respectively. The concept of literature-based discovery was introduced by Swanson [9] and has since been extended to many different areas of research. Several approaches have focused on mining both explicit associations based on co-occurrence, as well as implicit associations based on higher order co-occurrence and indirect relationships. CoPub Mapper [10] identifies shared terms that co-occur with gene names in MEDLINE abstracts. PubGene [11] constructs gene relationship networks based on co-occurrence of gene symbols in MEDLINE abstracts. Chilibot [12] is a Web-based system which extracts and characterizes relationships between genes, proteins and other terms. Wren *et al*. devised a method to calculate implicit association scores between biological entities and subsequently used it to functionally cluster genes [13,14].

Several IR approaches have focused on mining TF specific regulatory associations. Dragon TF association miner [15] is a Web-based tool that accepts as input a set of abstracts, and identifies and extracts TF associations with Gene Ontology terms found within the text. Šarić *et al*. (2006) and Rodriguez-Penagos *et al*. (2007) have used natural language processing to identify sentences pertaining to transcriptional regulation and extract relationships from PubMed abstracts to reconstruct regulatory networks [16,17]. More recent efforts have concentrated on novel TF discovery by analyzing protein mentions and related contextual information in literature to determine whether a given protein might be a TF [18].

Our group has applied various matrix factorization methods, such as Singular Value Decomposition (SVD), to extract functional relationships among genes from MEDLINE abstracts. SVD is a dimensionality reduction technique that decomposes the original term-by-document weighted frequency count matrix into a new set of factor matrices which can be used to represent both terms and documents in a low-dimensional subspace. Previously, we demonstrated that SVD can extract both explicit (direct) and implicit (indirect) relationships amongst genomic entities based on keyword queries, as well as gene-abstract queries, from the biomedical literature with better accuracy than term co-occurrence methods [19]. In this study, we have extended this approach to rank putative TFs for microarray derived differentially expressed gene sets. This study is unique in two ways. First, it applies SVD on a genome wide scale (~21K genes) using a large collection of abstracts (>650K). Second, it ranks and assigns p-values to TFs that may play a regulatory role for a subset of co-expressed genes.

## Methods

### Gene documents collection

For every gene, a gene abstract document was constructed by concatenation of all Medline titles and abstracts cross referenced in the Entrez Gene repository. The citations (identified by unique PubMed identifiers or PMIDs) are assigned either by professional staff at the National Library of Medicine or by the scientific research community via Gene Reference into Function (Gene RIF) portal. Since these abstracts are manually curated, we expect to have a very high precision for tagging correct abstracts to genes. It is important to note that the number of abstracts represented for each gene in the Entrez Gene repository is a small proportion of the total number of relevant abstracts in Medline for each gene, resulting in low recall. We further filtered the non-specific abstracts by removing PMIDs that referred to more than 10 genes as these citations usually described sequencing experiments mentioning a large number of genes in peripheral context but contained no significant functional information. After filtering, 21,027 mouse genes remained in the collection. The number of abstracts assigned to genes ranged from 1 (approximately 25% of the collection) to 5,396. The average and median number of abstracts in the collection were 32 and 5, respectively.

### Construction of LSI models

**Figure 1:**
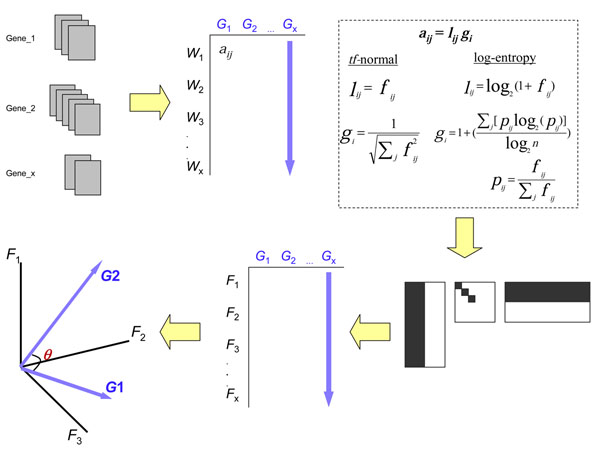
**Overview of the LSI based procedure to calculate association values between genes.** Gene documents were created for each of the 21,027 genes in the mouse genome by concatenating titles and abstracts corresponding to the genes. The documents were parsed to produce a term-by-gene matrix, the entries of which contained weighted term frequencies *a_ij_* calculated in two ways. The matrix was first normalized and then its dimensionality reduced using SVD. The association between any two genes was calculated as the cosine between any two gene document vectors in 500 dimensions.

The outline of the LSI approach used in this study is depicted in Figure 1. More than 400,000 terms (tokens) were parsed from the collection of gene documents using General Text Parser software [20]. All punctuation (excluding hyphens and underscores) and capitalization were ignored and, in addition, articles and other common, non-distinguishing words were discarded using the stoplist from Cornell's SMART project repository [21]. A term-by-gene matrix was created where the entries of the matrix were weighted frequencies of terms across the gene document collection. We explored two variants of term weighting schemes, term frequency normalization (*tf*-normal), and log-entropy normalization for building our two LSI models. Term weighting schemes are typically employed in order to normalize the matrix and discount the effect of common terms while at the same time increasing the importance of terms that are better delineators between gene documents. Each matrix entry *a_ij_* is transformed into a product of a local component (*l_ij_*) and global component (*g_i_*). For the *tf*-normal model:

and, for the log-entropy model:

where *f_ij_* is the frequency of the i^th^ term in the j^th^ gene-document, *p_ij_* is the probability of the i^th^ term occurring in the j^th^ gene-document and *n* is the number of gene documents in the collection. The *tf*-normal weighting scheme is useful in extracting explicit associations, whereas the log-entropy weighting scheme is based on information-theoretic concepts and takes into account the distribution of terms over gene-documents and is more useful in extracting implied relationships [22].

For both types of term weighting schemes, a reduced rank term-by-gene matrix was generated by computing the SVD as described in [19]. A rank of *k* = 500 was used to calculate the truncated matrix. Genes were then represented as vectors in the reduced rank matrix, and the association between any two genes was calculated as the cosine of the angle between the respective gene document vectors. The association scores can theoretically fall between -1 and 1, but in practice were observed to occur between 0-ε and 1 (ε << 0.01). A higher association score between a pair of genes indicates a stronger relationship in literature.

### Construction of co-occurrence model

In order to compare our LSI models against a literature-based benchmark, we devised and implemented a co-occurrence model. PMIDs for every gene (including the TFs) were obtained from the Entrez Gene repository as described above. An association score between any two genes was simply defined as the number of shared PMIDs between them.

### Calculation of TF literature enrichment p-values

In the literature models described above, a TF has an association score with every other gene. The goal of significance testing is to determine if the average literature association score for a TF with a given gene set is significantly higher than the average literature association score of that TF with a randomly selected set of genes.

For a given TF *t*, a target gene dataset *G*, and the entire gene population *P*,

Let,

t_G_ = {t_g_1_, t_g_2_, …, t_g_n_} be the set of association scores between the TF *t* and genes in the gene dataset *G*. n is the number of genes in *G*.

s = standard deviation of t_G_

t_P_ = {t_g_1_, t_g_2_, …, t_g_N_} – { t_g_t_} be the set of association scores between the TF *t* and all other genes in the population *P*. N is the total number of genes in *P*. The association score of TF *t* with itself is excluded.

µ = mean of t_P_

To calculate the TF enrichment p-value, we conducted a right tailed one sample Student’s t-test [23] between the set t_G_ and µ with a significance level (alpha) of 0.05. The p-value is the probability, under the null hypothesis, of observing a value as extreme or more extreme of the test statistic

A TF that has higher average literature-based association with a target gene set relative to the entire gene population is deemed more significant than a TF that does not.

### Datasets

To evaluate our algorithms, five published microarray datasets were chosen from Gene Expression Omnibus (GEO) [24] available from the National Center for Biotechnology Information (NCBI) [25]. Each experiment examined gene expression for untreated and treated conditions. Importantly, each experiment was designed to investigate the role of a specific TF in mediating the effect of the stimulation on gene expression changes. As shown in Table 1, the datasets focused on TFs *Rel *[26], *Stat6*[27], *Ddit3*[28], *Stat5*[29] and *Nfic *[30]. We used these TFs as ground truth to evaluate the performance of our methods. The list of co-expressed genes for each experiment is presented in Supplementary table 1 in additional file 1.

**Table 1:** Datasets used for evaluation of LSI based methods.

Dataset No.	GEO Series	Stimulant	TF Knockout	# DEGs (n)
**1**	GSE3400	Interferon	*Rel*	95
**2**	GSE20030	IL-4	*Stat6*	50
**3**	GSE2082	Tunicamycin	*Ddit3* (*CHOP*)	55
**4**	GSE21861	Growth Hormone (GH)	*Stat5a/b*	61
**5**	GSE15871	TGF-β1	*Nfic*	51

### Construction of gold standard TFs

As a second approach to evaluate our methods, we constructed a set of gold standard TFs for each microarray dataset by manually analyzing the published literature. The goal here was to connect the type of stimulation (cell signaling pathway) to the TFs by identifying experimentally supported statements in published literature. First, we used a Web-based NLP tool Chilibot [12] to identify abstracts and sentences that were shared between all TFs and the specific stimulant used in the study. Then, each sentence was manually inspected to confirm the interaction between the TFs and the stimulant. A TF was said to be directly associated with a stimulant if there was at least one sentence providing experimental support for their interaction. This process led to the identification of 209, 148, 42, 139 and 257 relevant TFs for Interferon, IL-4, Tunicamycin, Growth Hormone and TGF-β1 datasets, respectively. Supplementary table 2 in additional file 1 includes the list of all gold standard TFs manually constructed for each dataset.

### Workflow

**Figure 2:**
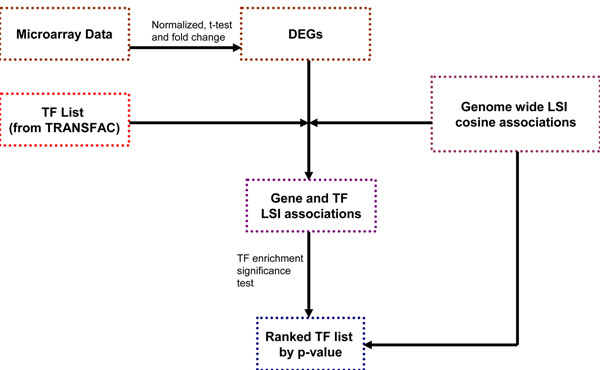
**Workflow for the LSI based TF ranking for microarray derived gene sets.** Microarray data was analyzed to identify differentially expressed genes (DEGs) in response to treatments. A list of 433 mouse TFs was derived from the TRANSFAC database and a significance test was conducted to identify TFs showing high average literature association with the entire set of DEGs relative to the entire gene population of 21,027 genes. TFs were ranked according to the literature-derived enrichment p-values.

Figure 2 outlines the workflow of our method to rank putative TFs for a given microarray experiment. Gene expression data were preprocessed, normalized and subjected to a Welch’s t-test [31] to identify differentially expressed genes which showed greater than 2-fold change between stimulated and un-stimulated conditions. Literature associations between the DEGs and all 433 TFs annotated in TRANSFAC were determined using two different LSI models as well as a co-occurrence model described above. To calculate the p-value for a TF association with the observed DEGs, we performed a right-tailed Student’s t-test comparing the TF association scores with the DEGs to the mean of the TF association scores with the entire gene population. The p-values were used to rank each TF and to determine which ones had the most significant literature association to the majority of the observed DEGs for a given experiment.

## Results

### TF ranking using LSI based association scores

The goal of our study was to identify TFs that play critical regulatory roles in mediating gene expression changes induced by signaling molecules. These TFs may regulate gene expression directly via binding to gene promoters or indirectly via regulation of other TFs. Current methods rely on motif searching approaches, which at best will identify direct TF-gene associations. Another challenge with these approaches is that many motifs exist in gene promoters and multiple TFs may bind to a specific motif, thus it is difficult to prioritize which motifs may play a functionally important role for a set of DEGs. For instance, using Web-based motif searching tool CORE_TF we identified 86 overrepresented motifs for a set of Interferon stimulated genes, corresponding to 125 different TFs, with an average of 2.55 TFs per motif (Table 2).

**Table 2:** CORE_TF motif ranking for five microarray derived gene sets.

	Core_TF Results		
	
	# motifs (p-value = 0)	Avg # TFs per motif	Total # of TFs
**Interferon stimulated genes**	86	2.55	125
**IL-4 stimulated genes**	10	1.60	16
**Tunicamycin stimulated genes**	5	1.20	6
**Growth Hormone (GH) stimulated genes**	27	2.26	40
**TGF-β1 stimulated genes**	7	2.29	10

Multiple TF motifs were ranked first (p-value=0) for the various gene sets. Also, each TF motif was mapped to multiple TFs, making it difficult to prioritize critical TFs for each gene set.

**Figure 3:**
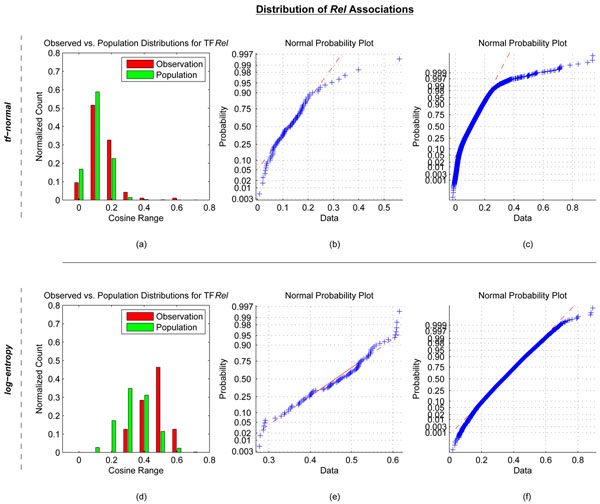
**Distribution of LSI based association scores for TF *Rel* in the Interferon dataset.** Results of the *tf*-normal (a-c), and log-entropy (d-f) LSI models. (a) and (d) Histograms of cosine distributions of TF *Rel* with the set of 95 DEGs responding to Interferon stimulation (observed set, red bars) as well as the entire set of 21,027 genes in the mouse genome (excluding *Rel*) (population set, green bars). (b) and (e) Normality plot of distribution of cosines for the observed *Rel* associations. (c) and (f) Normality plot of distribution of cosines for all *Rel* associations.

To help prioritize functionally relevant TFs for a set of DEGs, we utilized LSI to extract associations between TFs and sets of DEGs using the information in Medline abstracts. Two different term-weighting schemes were used. As mentioned earlier, the *tf*-normal weighting scheme is useful in extracting explicit associations, whereas the log-entropy weighting scheme is more useful in extracting implied relationships. To determine if the TF-gene associations identified by these models were significant, for each TF, we compared the TF association scores with the observed set of DEGs to the mean of the TF association scores with the entire gene population (consisting of >21,000 genes), using a right-tailed one sample t-test. For both LSI models, we found that that the association scores were normally distributed for the vast majority of TFs. As an example, Figure 3 shows the distribution of LSI association scores for TF *Rel* with the set of Interferon induced DEGs compared to the scores observed for the entire gene population. The range of association scores in the *tf*-normal LSI model is less than the range of association scores in the log-entropy LSI models. For both models, the distribution of *Rel* association scores with the Interferon stimulated DEGs was skewed to the right of the population distribution. This indicates that *Rel* has higher association in literature with the set of Interferon stimulated DEGs than with a random set of genes derived from the population. Furthermore, we investigated the normality of the distribution of *Rel* association scores. We found that *Rel* association scores with either Interferon DEGs or the entire gene population were normal for the log-entropy model (Figure 3, e and f) but somewhat skewed for the *tf*-normal model (Figure 3, b and c). Similar trends were observed for the other TFs and datasets.

**Figure 4:**
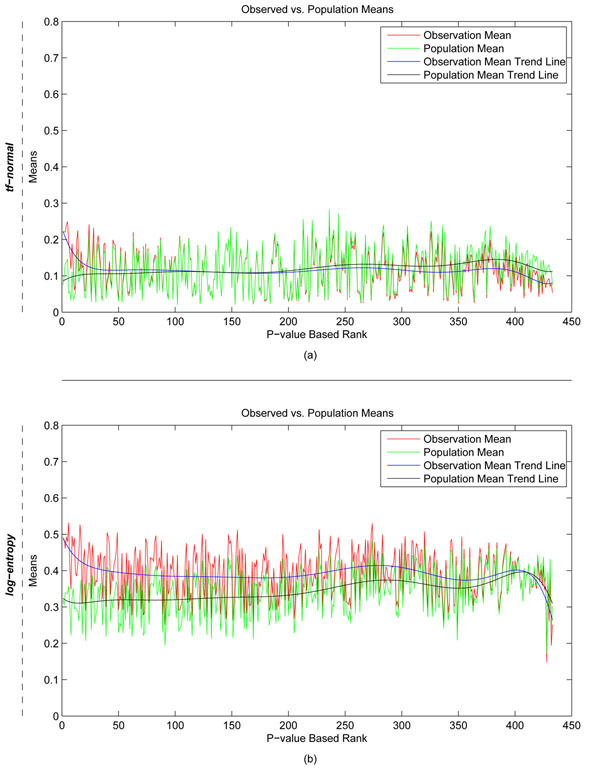
**Correlation between cosine scores and TF enrichment p-values for the Interferon dataset.** Average cosine values derived by **(a)***tf*-normal LSI model or **(b)** log-entropy LSI model between all 433 TFs and 95 Interferon DEGs (red line) or the entire population of 21,027 genes (green line).

Using the procedure described above, a p-value was generated for each of the 433 TFs with respect to literature association with the DEGs. We posit that the most relevant TF is the one with the highest association, hence lowest p-value. Figure 4 shows the correlation between TF enrichment p-values and mean association scores for all 433 TFs with respect to the observed Interferon stimulated DEGs (red) or the entire gene population (green). As expected, we found that the difference between the observed and population means decreased as a function of increasing p-values. We also found that this difference rapidly dropped with increasing p-values for the *tf*-normal model compared with the log-entropy model. This indicates that fewer TFs are deemed significantly associated with the DEGs according to the *tf*-normal (more explicit) model than the log entropy model.

### Evaluation of TF rankings

The top 25 ranked TFs for each of the five microarray datasets using either the *tf*-normal or log-entropy LSI models are displayed in Tables 3 and 4. To test the performance of each model we used multiple approaches. First, we compared the rankings of the TFs that were specifically targeted in each study. For instance, *Rel* was treated as a gold standard in our study because the original study investigated the role of *Rel* in Interferon induced gene expression in fibrobasts from *Rel* knock-out mice compared with wild-type controls [26,32]. Similarly for the other datasets, transcription factors *Stat6* (IL-4 signaling), *Ddit3* (Tunicamycin response), *Stat5* (Growth Hormone signaling), and *Nfic* (TGF-β1 signaling) were investigated respectively [27-30]. Interestingly, 4 of the 5 TF targets (*Rel*, *Stat6*, *Ddit3* and *Stat5*) were ranked amongst the top 25 TFs ranked by the *tf*-normal model compared to two (*Rel* and *Stat6*) ranked by the log-entropy model.

**Table 3:** Top 25 ranked TFs for five microarray derived gene sets using *tf*-normal LSI model.

*tf*-normal LSI model					
**Rank**	**Interferon**	**IL-4**	**Tunicamycin**	**GH**	**TGF-**β**1**

**1**	*Irf7*	*Stat4*	*Yy1*	*Nfkb1*	*Smad7*
**2**	*Irf1*	***Stat6***	*Atf4*	*Nfkb2*	*Smad9*
**3**	*Irf8*	*Irf1*	*Zbtb7a*	*Rela*	*Postn*
**4**	*Irf5*	*Irf5*	*Atf7*	*Rel*	*Smad6*
**5**	*Irf2*	*Stat1*	*Nfyb*	*Ifi47*	*Smad1*
**6**	*Irf3*	*Ifi47*	*Zfp143*	*Stat4*	*Smad5*
**7**	*Irf9*	*Nfkb1*	*Nfya*	*Irf7*	*Hif1a*
**8**	*Stat1*	*Itgal*	*Zbtb6*	*Stat6*	*Nfkb1*
**9**	*Ifi47*	*Stat2*	*Atf2*	*Irf1*	*Stat3*
**10**	*Stat2*	*Irf3*	*Elk1*	*Stat1*	*Egr1*
**11**	*Stat4*	*Irf7*	*Nfyc*	*Stat2*	*Nfatc1*
**12**	*Nfkb1*	*Pparg*	*Cebpe*	*Stat3*	*Smad4*
**13**	*Irf4*	*Irf9*	*Atf5*	***Stat5a***	*Smad3*
**14**	*Zfp143*	*Foxp3*	*Junb*	*Cebpb*	*Smad2*
**15**	*Nfkb2*	*Irf8*	*Atf6*	*Irf3*	*Runx2*
**16**	*Cebpe*	*Nfe2l2*	*Bach1*	***Stat5b***	*Stat5a*
**17**	*Zbtb6*	*Stat3*	*E4f1*	*Irf9*	*Gata4*
**18**	*Elf4*	*Foxo1*	*Elk4*	*Foxp3*	*Tgif1*
**19**	*Ddit3*	*Irf2*	*Atf3*	*Foxo3*	*Foxm1*
**20**	*Stat3*	*Ppard*	*Zfp148*	*Irf5*	*Gata6*
**21**	*Rela*	*Ppara*	***Ddit3***	*Itgal*	*Gata5*
**22**	***Rel***	*Foxo3*	*Jund*	*Irf8*	*Nkx2-5*
**23**	*Atf4*	*Srebf1*	*Hsf2*	*Cebpe*	*Lef1*
**24**	*Xbp1*	*Srebf2*	*Elk3*	*Pparg*	*Cebpd*
**25**	*Hsf1*	*Ddit3*	*Hsf1*	*E2f1*	*Nkx3-2*

TFs displayed in bold font were used as ground truth because they were targeted in the published study as critical regulators for each gene set.

**Table 4:** Top 25 ranked TFs for five microarray derived gene sets using log-entropy LSI model.

log-entropy LSI model					
**Rank**	**Interferon**	**IL-4**	**Tunicamycin**	**GH**	**TGF-β1**

**1**	*Irf8*	*Stat3*	*Nfyc*	*Irf1*	*Gata4*
**2**	*Irf1*	*Nfkb1*	*Nfyb*	*Stat1*	*Gata6*
**3**	***Rel***	*Smad3*	*Nfya*	*Stat6*	*Wt1*
**4**	*Irf5*	*Stat1*	*Zbtb7a*	*Stat4*	*Cdx2*
**5**	*Irf4*	*Rela*	*Nfe2l1*	*Nfe2l2*	*Tcfap2a*
**6**	*Irf2*	*Egr1*	*Zfp143*	*Egr1*	*Fosl1*
**7**	*Nfkb2*	*Jun*	*Hsf2*	*Creb1*	*Postn*
**8**	*Stat1*	*Stat5a*	*Rfx5*	*Smad3*	*Smad1*
**9**	*Stat2*	*Pparg*	*Maz*	*Hif1a*	*Pgr*
**10**	*Prdm1*	*Irf1*	*Atf7*	*Hp*	*Egr1*
**11**	*Irf7*	*Foxo3*	*Zfp148*	*Sfpi1*	*Sox9*
**12**	*Nfatc2*	*Vdr*	*Tcfap4*	*Sp1*	*Srf*
**13**	*Sfpi1*	*Smad7*	*Cebpg*	*Stat3*	*Smad7*
**14**	*Stat4*	*Kitl*	*Mafg*	*Nr3c1*	*Arnt*
**15**	*Irf3*	*Sp1*	*Rfx1*	*Irf3*	*Smad3*
**16**	*Irf9*	***Stat6***	*Sp2*	*Smad7*	*Gli1*
**17**	*Gfi1*	*Hif1a*	*Gtf2i*	*Cebpb*	*Pax8*
**18**	*Rela*	*Stat5b*	*Bach1*	*Irf5*	*Rarg*
**19**	*Bcl6*	*Fos*	*Gabpb1*	*Fos*	*Ar*
**20**	*Xbp1*	*Gata3*	*Tcfcp2*	*Irf8*	*Smad2*
**21**	*Nfkb1*	*Stat4*	*E4f1*	*Kitl*	*Tcf7l2*
**22**	*Nfatc3*	*Myc*	*Bach2*	*Itgal*	*Nkx2-1*
**23**	*Atf6*	*Nr3c1*	*Elf2*	*Ahr*	*Foxo1*
**24**	*Atf3*	*Foxp3*	*Mxd1*	*Gata1*	*Gata3*
**25**	*Cebpe*	*Esr1*	*Mxd4*	*Pparg*	*Lef1*

TFs displayed in bold font were used as ground truth because they were targeted in the published study as critical regulators for each gene set.

**Figure 5:**
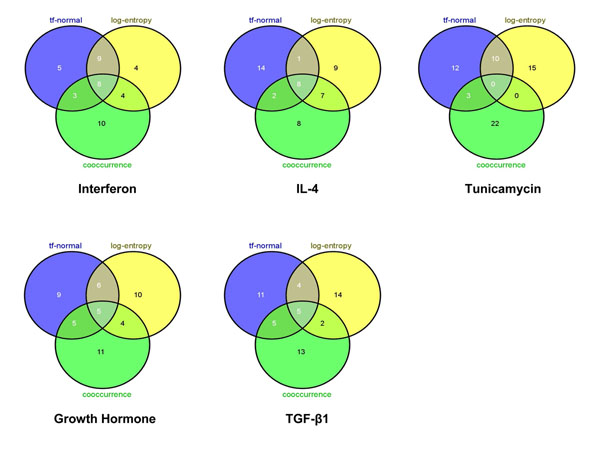
**Overlap between top 25 ranked TFs derived via *tf*-normal, log-entropy and cooccurrence models.** (Venn diagrams generated with Web-tool VENNY [37])

Since both LSI based text-mining approaches performed reasonably well, we asked if they outperformed simple co-occurrence approaches. Here, we simply scored an association between a TF and the target genes by the number of abstracts they shared among those manually curated in the Entrez Gene repository. Importantly, only one TF (*Ddit3*) was identified in the top 25 ranked TFs for the 5 different datasets (Table 5). A comparison of the results from the three different text-based approaches showed that there is considerable overlap between the two LSI models and the co-occurrence model for some datasets, e.g., Interferon and IL-4 (Figure 5). In contrast, there was no overlap between the TFs identified by the three different models for Tunicamycin dataset. Interestingly, for this dataset, the co-occurrence model identified the candidate TF to be ranked first. This result indicates that in general the co-occurrence based method performed poorly, but in the case of *Ddit3*, it performed better than both LSI models (Table 5).

**Table 5:** Top 25 ranked TFs for five microarray derived gene sets using the abstract cooccurrence model.

abstract co-occurrrence model					
**Rank**	**Interferon**	**IL-4**	**Tunicamycin**	**GH**	**TGF**-β**1**

**1**	*Irf1*	*Hif1a*	***Ddit3***	*Nfkb1*	*Hif1a*
**2**	*Stat1*	*Sfpi1*	*Egr1*	*Stat1*	*Egr1*
**3**	*Irf9*	*Tcfap2a*	*Gfi1*	*Cebpb*	*Runx2*
**4**	*Stat6*	*Irf1*	*Ppara*	*Rel*	*Smad3*
**5**	*Stat4*	*Rela*	*Ppard*	*Foxp3*	*Sp1*
**6**	*Nr3c1*	*Egr1*	*Tcfap2a*	*Kitl*	*Smad4*
**7**	*Irf3*	*Pparg*	*Tcf3*	*Nfatc2*	*Smad1*
**8**	*Sfpi1*	*Nfkb1*	*Rara*	*Rela*	*Tcf3*
**9**	*Myc*	*Stat1*	*Zbtb16*	*Irf1*	*Nkx2-5*
**10**	*Jund*	*Ikzf1*	*Rarg*	*Stat6*	*Tcf4*
**11**	*Atf3*	*Foxo1*	*Cebpd*	*Jun*	*Elk3*
**12**	*Ifi47*	*Gata1*	*Hoxa9*	*Sfpi1*	*Tcfap2a*
**13**	*Prdm1*	*Itgal*	*Rxra*	*Cebpd*	*Tcf12*
**14**	*Stat3*	*Kitl*	*Hsf1*	*Ddit3*	*Smad7*
**15**	*Foxn1*	*Foxn1*	*Nr5a1*	*Foxn1*	*Zbtb16*
**16**	*Ep300*	*Fosl1*	*Pparg*	*Irf3*	*Bcl6*
**17**	*Usf1*	*Stat6*	*Otx1*	*Ep300*	*Pgr*
**18**	*Irf8*	*Stat3*	*Ep300*	*Vdr*	*Cebpd*
**19**	*Bcl6*	*Fos*	*Jund*	*Cebpa*	*Ep300*
**20**	*Rxrb*	*Jun*	*Srebf1*	*Fos*	*Smad2*
**21**	*Hsf1*	*Nfatc2*	*Hand1*	*E2f1*	*Jun*
**22**	*Rela*	*Foxp3*	*Hif1a*	*Sp1*	*Foxo3*
**23**	*Junb*	*Sp1*	*Egr2*	*Ppara*	*Pax5*
**24**	*Fos*	*Foxo3*	*Runx2*	*Myb*	*Ppara*
**25**	*Stat2*	*Pxn*	*Gata3*	*Pax5*	*Myod1*

*Ddit3* (in bold font) was the only ground truth TF ranked by this method. The ground truth TFs were chosen because they were targeted in the published study as critical regulators for each gene set.

We also compared our results with those from a Web-based motif searching tool CORE_TF [1]. This tool determines motif overrepresentation p-values in the promoter regions of a given gene set, using 525 vertebrate motif definitions in TRANSFAC database version 11.2. We found that multiple motifs shared the same p-values, making it difficult to rank TFs. Also, motifs were associated with multiple TFs and a given TF was associated with multiple motifs (Table 2). For our evaluation, we chose the motif for a TF of interest that had the lowest p-value in the CORE_TF ranking. Table 6 compares the rankings produced by CORE_TF with those produced by the three literature-based models. We observe that in the case of IL-4 (*Stat6*), Tunicamycin (*Ddit3*) and possibly Interferon (*Rel*), both LSI models performed better than CORE_TF, whereas the three approaches produced similar results for TGF-β1 (*Nfic*) and Growth Hormone (*Stat5*). Only in the case of Tunicamycin dataset, the co-occurrence model seemed to outperform the other three methods.

**Table 6:** Comparison of TF rankings produced by four different methods for the five datasets.

Dataset	TF knockout	*tf*-normal	log-entropy	co-occurrence	CORE_TF
**Interferon**	*Rel*	22	3	90	[1--86]^*^
**IL-4**	*Stat6*	2	16	17	69
**Tunicamycin**	*Ddit3*	21	128	1	-^†^
**GH**	*Stat5a/b*	13	50	29	9
**TGF**-β**1**	*Nfic*	241	233	[205--433]^§^	289

^*^Using CORE_TF, *Rel* could be ranked anywhere between 1 and 86 as its associated motif V$CREL_01 had a p-value = 0 (rank 1) along with 85 other motifs. ^†^The motif for *Ddit3*, V$CHOP_01 was not ranked by CORE_TF. ^§^Using the abstract co-occurrence model, the TF *Nfic* could be ranked anywhere between 205 and 433 as it did not share any abstracts with any gene in the TGF-β1 dataset and therefore had a p-value of 1 along with 228 other TFs.

**Figure 6:**
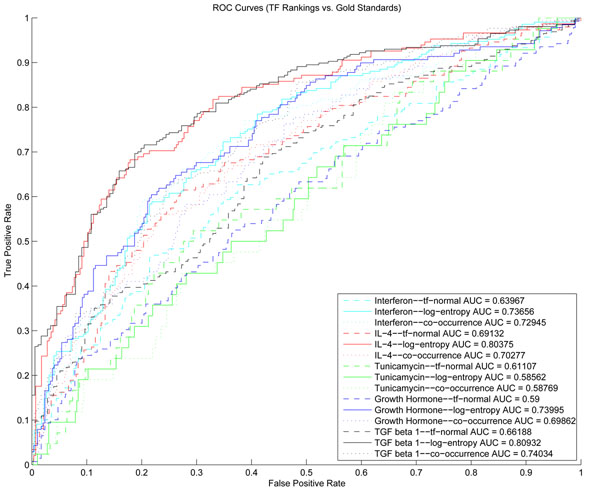
**ROC curves for the TF rankings produced by three literature-based models for five datasets.** The TF gold standards were determined by manual examination of experimental evidence as reported in PubMed.

Lastly, since there were no well-defined gold standards for evaluation of our methods, and using singleton TFs as gold standards does not constitute a thorough evaluation of a ranking, we manually constructed gold standard TFs for each dataset by analyzing the published literature. We evaluated our TF rankings against these gold standards by generating Receiver Operating Characteristics (ROC) curves which display recall and false positive rates at each rank (Figure 6). The area under the curve (AUC) can be used as a measure of ranking quality [33,34]. The AUC will have the value of 1 for perfect ranking (all relevant TFs at the top), 0.5 for randomly generated ranking, and 0 for the worst possible ranking (all relevant TFs at the bottom). Importantly, except for the Tunicamycin dataset, all AUC values produced by the three models were substantially higher than the chance value of 0.5. Interestingly, in all four cases, the log-entropy LSI model achieved the highest AUC values (ranging between 0.73 and 0.81) compared to *tf*-normal and co-occurrence models. Tunicamycin dataset produced very low AUC values for all three models. One reason for the low performance of all three models for this dataset could be that only 42 TFs out of 433 (~9 %) were designated as gold standard. We attribute the ability of the log-entropy model to pull out implicit associations via text for its consistent high performance across the four datasets.

It is important to point out that more than 50% of the 433 TFs did not co-occur with a gene in the different datasets. The TF-gene co-occurrence rates for Interferon, IL-4, Tunicamycin, Growth Hormone, and TGF-β1 datasets were 40%, 36%, 31%, 38%, and 48%, respectively. For all these TFs, the p-values obtained via the co-occurrence model were 1 because the associations were all zeros. Consequently, the ranking of these TFs may be arbitrary and difficult to interpret. In contrast, the LSI based models can rank TFs irrespective of whether or not they co-occur with any gene in the target gene set.

## Discussion

We have developed an LSI based approach to identify potentially important transcription factors in a gene regulatory network from gene expression datasets. The underlying hypothesis of our approach is that a TF plays a critical role in mediating the effects of cell signaling stimulation if it has functional association with the majority of the DEGs induced by the specific stimulation. Because direct experimental information about TF and gene interactions is limited in the biomedical literature, we have explored the use of LSI based text-mining approach that can extract both explicit and implicit associations from the literature. We compared two different term-weighting schemes in the LSI models against a standard motif searching algorithm as well as a co-occurrence based approach. In general, our method performed well and could provide a complementary tool for investigating gene regulatory networks (Table 6).

It has been difficult to identify a true gold standard to measure the performance of our approach. Our first approach used the targeted TF in the microarray experiment as a gold standard. In these experiments, the authors hypothesized that a TF was involved in mediating the expression of a set of genes, thus examined DEGs in TF knockout cells compared to normal controls. This is a useful gold standard as it identifies TFs that are both directly and indirectly associated with the DEGs. However, we found that the chosen TFs were truly hypothetical and some of them were remotely associated with the signaling pathway under study. Also, the TF *Nfic* was not ranked high by either of the LSI models even though it scored a high average cosine with the gene set (data not shown) and has explicit association with TGF-β1. Our ranking scheme gives priority to TFs that score a high average cosine with the target gene set relative to the entire gene population. Notably, *Nfic* scored relatively high with the population as well, resulting in a larger p-value. It appears that *Nfic* might be a more generic TF associated with many genes and thus not very specific to our target gene set. Importantly however, our method identified many TF targets that were higher ranked than the singleton TF that was targeted in the microarray study (Tables 3 and 4).

To test the overall performance of our method, we had to manually construct a new set of gold standard TFs for each microarray experiment. There are a number of ways that gold standards could have been generated. The most popular methods rely on curated databases that contain certain biochemical or interaction data. However, these databases would not be appropriate for evaluation of our specific methods which aim to identify direct and indirect regulation of genes by TFs. For instance, information in pathway interaction domains (PID) would only inform about TF-TF interactions. Gene Ontology (GO) and Kyoto Encyclopedia of Genes and Genomes (KEGG) have limited information about specific pathways. Alternatively, our text-mining method could be enhanced by including TF binding sequences and their association with genes from the biomedical literature [35]. However, motif sequences are rarely presented in abstracts and, therefore, would require us to access full text articles which are not freely available. Lastly, Gene-TF interaction data could be acquired by Chromatin IP-chip experiments. However, these only provide direct TF-gene interaction data and would not reveal indirect regulation of gene expression. Therefore, we resorted to analyzing published experiments available in Medline to cull the gold standard TFs for each dataset. The rationale for this approach was that for each experiment, the stimulant of interest elicited changes in the expression of a set of genes. If TFs are accurately associated with the gene set by our models, then we expect independent experimental evidence that links the stimulant to the TFs. In other words, we are testing whether the TF-gene associations are consistent with the TF-stimulant associations in the literature.

Based on the ROC results, we suggest that in general the log-entropy LSI model performs better than *tf*-normal and co-occurrence models, albeit with varying degrees (Figure 6). In one case (Tunicamycin dataset), the *tf*-normal model outperformed the log-entropy model and cooccurrence model. There are two possible explanations for the poor performance of the cooccurrence model in nearly all datasets. First, since the associations here are based on the number of shared abstracts between TF and genes, more than half of all TFs did not co-occur with any gene. This distribution is not appropriate for the p-value calculations. Second, the low abstract counts may be due to low overall recall of relevant abstracts tagged to the genes by the Entrez Gene curators. While this highlights a potential disadvantage of using human curated gene abstracts, it is advantageous for LSI modeling. Because in LSI models, gene associations are based on word usage patterns, having high precision in gene abstracts is better than high recall. On the hand, high recall is preferred for co-occurrence methods because the more abstracts you can assign to a gene the higher the likelihood of finding co-occurrences. Another explanation for the poor performance on the Tunicamycin dataset may be that the microarray experiment itself was problematic and resulted in erroneous set of DEGs. It is important to note that we applied standard normalization and statistics to identify DEGs. It may have been better to use more robust normalization methods or other statistical tests.

Our LSI based method identifies new (implied) relationships that have not been explicitly described in the literature. This ability is particularly advantageous for discovery oriented genomic experiments, which aim to expose new associations. However, our evaluation procedure included only 'known' TF associations, which does not fully test the method's predictive value. Also, it is worth noting that the LSI associations (cosines) between TFs and genes may not be necessarily transcriptional in nature, as the cosine value is a weighted combination (both additive and subtractive) of several direct (explicit) and indirect (implicit) relationships, a large fraction of which may be biochemical pathway or signaling associations. Nonetheless, our method can identify possible TF targets which can then be tested experimentally. Another important advantage of our method is that it contains abstracts for 1260 of the approximately 1675 mouse transcription factors reported by RIKEN [36], in contrast to motif searching methods which contain 400-600 validated transcription factor motifs. Finally, our method can easily be adapted to rank other molecular associations, such as miRNA-gene or drug-gene associations using the biomedical literature.

## Conclusions

Taken together, we have developed a text-mining approach that can help systems biologists identify critical regulatory TFs from a set of co-regulated genes identified by microarray experiments. Using either the log-entropy or the *tf*-normal model, investigators can search for TFs which are either implicitly or explicitly associated with the DEGs and the cellular stimulation. These methods can nicely complement existing approaches that identify TF binding motifs in promoters of co-regulated genes. Our future efforts will focus on developing a Web-tool which will allow researchers to compare multiple text-mining models for any given gene set.

## Competing interests

RH, KH, and MWB are equity holders in Computable Genomix.

## Authors' contributions

SR and RH designed the research and wrote the manuscript. SR and KH wrote the implemented software. VP, MWB and RH supervised the research and assisted with interpretation of results.

## Supplementary Material

Additional file 1 • Supplementary Table 1: DEGs for five microarray datasets used in the study. • Supplementary Table 2: Manually assigned gold standard TFs directly associated with five different stimulants in published literature. Click here for file
