# Supplementary material for: Latent Semantic Indexing of PubMed abstracts for identification of transcription factor candidates from microarray derived gene sets
Source: BMC Bioinformatics. 2011 Oct 18;12(Suppl 10):S19. doi: 10.1186/1471-2105-12-S10-S19 (PMC3236841; doi:10.1186/1471-2105-12-S10-S19)
Supplement: Additional file 1 — • Supplementary Table 1: DEGs for five microarray datasets used in the study. • Supplementary Table 2: Manually assigned gold standard TFs directly associated with five different stimulants in published literature. [file 1471-2105-12-S10-S19-S1.pdf]

Supplementary Information

Supplementary Table 1. DEGs for five microarray datasets used in the study.

List of DEGs				
**Interferon**	**IL-4**	**Tunicamycin**	**GH**	**TGF-β1**
Isg15	*9030425E11Rik*	*Dnajc3*	*Apol7c*	*Mex3b*
Blmh	*Arg1*	*Zbtb48*	*Birc3*	*Adm*
D17H6S56E-5	*Arhgap18*	*Adm*	*Bcl2a1a*	*Alcam*
Aim1	*Arrdc3*	*Apobec1*	*Bcl3*	*Cdk5r1*
Ankfy1	*Ass1*	*Pdia4*	*Bcl2l11*	*Col3a1*
Apobec1	*Ccl24*	*Cbx4*	*C3*	*Epha3*
Apod	*Cd14*	*Clcn3*	*Cd14*	*Fbln2*
Casp12	*Cd28*	*Clk4*	*Cd69*	*Fgf18*
Socs1	*Cd93*	*Gadd45a*	*Cd83*	*Ctgf*
Cyp26a1	*Cerkl*	*Dmp1*	*Ch25h*	*Foxc2*
Daxx	*Cxcl10*	*Gas1*	*Cish*	*Foxs1*
Gbp2	*Cxcr4*	*Gcnt2*	*Socs3*	*Hbegf*
Gnb4	*Ear11*	*Nr4a1*	*Dusp2*	*Nr4a1*
H2-D1	*Emr4*	*Hoxa1*	*Gem*	*Id1*
H2-K1	*Entpd1*	*Elovl6*	*Cxcl1*	*Id2*
H2-L	*F10*	*Ppp1r15a*	*Icam1*	*Id3*
H2-T10	*Fcgr4*	*Gadd45b*	*Cxcl10*	*Id4*
H2-T22	*Flt1*	*Piga*	*Il12b*	*Ier3*
Htr1d	*Fn1*	*Pim1*	*Il1a*	*Ier5*
Irgm1	*Fpr1*	*Ptger4*	*Il1b*	*Cyr61*
Cxcl10	*Gpc1*	*Rad52*	*Irg1*	*Lif*
Ifi203	*Ifit2*	*Sel1l*	*Hivep3*	*Gadd45b*
Ifi204	*Ifit3*	*Siah2*	*Mup2*	*Nedd9*
Ifit1	*Ifitm6*	*St6gal1*	*Nfkbia*	*Nrp1*
Ifit2	*Igfbp4*	*Snai2*	*Nfkbie*	*Tnfrsf11b*
Ifit3	*Il1b*	*Taf1a*	*Pde4b*	*Pim1*
Igtp	*Il4i1*	*Zfp354a*	*Phf21a*	*Serpine1*
Il13ra1	*Itga4*	*Zfp322a*	*Relb*	*Ptgs2*
Il6	*Itga9*	*Kdm6a*	*Saa3*	*Sgk1*
Cd47	*Itgax*	*Uhrf1bp1*	*Ccl2*	*Skil*
Jak2	*Mgl2*	*Zfp105*	*Ccl3*	*Slc20a1*
Lgals9	*Mmp9*	*Zfp30*	*Ccl4*	*Snai1*
Psmb8	*Pdcd1lg2*	*Zfp51*	*Ccl5*	*Csrnp1*
Ly6e	*Pde3b*	*Zfp60*	*Ccl7*	*Wnt9a*
Mov10	*Hpgds*	*Zfp61*	*Cxcl2*	*Klf10*
Mx1	*Rcbtb2*	*Zscan12*	*Cxcl5*	*Zfp503*
Mx2	*Retnla*	*Zfp97*	*Cx3cl1*	*Spata13*

Myd88	*Rnf144b*	*Zfpm2*	*Slfn2*	*Gadd45g*
Plscr2	*Saa3*	*Rabepk*	*Nlrp3*	*Ppp1r3c*
Pml	*Scd2*	*Trib3*	*Tnf*	*Map3k14*
Lgals3bp	*Sepp1*	*Dnajb9*	*Tnfaip2*	*Slc40a1*
Eif2ak2	*Slc40a1*	*Sfrs17b*	*Tnfaip3*	*Txnip*
Psmb10	*Slc7a11*	*Txnip*	*Tnfrsf1b*	*Pmepa1*
Psme1	*Slc7a2*	*Herpud1*	*Cd40*	*1190002H23Rik*
Serpinb9	*Stk17b*	*Zfp110*	*Tnfsf9*	*2810474O19Rik*
Trim21	*Thbs1*	*Trappc5*	*Slc2a6*	*Rasl11b*
Stxbp1	*Tlr2*	*Trim59*	*Zc3h12a*	*Bcor*
Tap1	*Tlr7*	*Rnmtl1*	*Malt1*	*Nuak2*
Tap2	*Tm7sf4*	*Ero1lb*	*Tlr2*	*Rnd3*
Tapbp	*Wwp1*	*Ccdc130*	*Map3k8*	*Spsb1*
Zcchc6		*Phkg2*	*Cxcl3*	*Mfsd2a*
Trim25		*Taf1d*	*Ccrl2*	
Tgtp1		*Creld2*	*Parp14*	
Xdh		*Bat4*	*Gbp3*	
AI607873		*Snhg1*	*Plek*	
Ifi205			*Clec4e*	
Gbp6			*Stx11*	
Trafd1			*Igsf6*	
Gadd45g			*Nfkbiz*	
Tor1aip2			*Niacr1*	
Usp18			*Gpr84*	
Ifi202b				
Tcirg1				
Azi2				
Ddx24				
Tor3a				
Sfmbt2				
Nub1				
Irgm2				
Gbp3				
Samhd1				
Akap8				
Adar				
Ube2l6				
Isg20				
Rsad2				
Nampt				
Iigp1				
Psme2b-ps				
Nmi				

Ifitm3
Isoc1
Peli1
5-Mar
Ifi35
Ifih1
Pnpt1
Ogfr
Uba7
Ftsjd2
Rnf19b
Etnk1
Ccdc6
N4bp1
Znfx1

Supplementary Table 2. Manually assigned gold standard TFs directly associated with five different stimulants in published literature.

List of gold standard TFs				
**Interferon**	**IL-4**	**Tunicamycin**	**GH**	**TGF-β1**
Irf9	*Stat6*	*Atf6*	*Pou1f1*	*Smad6*
Irf2	*Nfatc2*	*Ddit3*	*Lhx3*	*Smad7*
Irf1	*Stat4*	*Xbp1*	*Sox3*	*Tgif1*
Irf3	*Bcl6*	*Atf4*	*Nr5a2*	*Postn*
Irf7	*Gfi1*	*Cebpg*	*Nr2f2*	*Smad2*
Stat2	*Nfatc3*	*Cebpb*	*Nr5a1*	*Msx1*
Irf8	*Nfatc1*	*Cebpa*	*Nfya*	*Smad3*
Ifi47	*Maf*	*Hp*	*Pcbp1*	*Sox17*
Irf5	*Gata3*	*Hlf*	*Nfic*	*Smad9*
Stat1	*Irf1*	*Rfx1*	*Tff3*	*Smad4*
Zfp143	*Tff3*	*Gabpa*	*Sf1*	*Foxh1*
Rfx5	*Irf5*	*Nrf1*	*Esr1*	*Smad5*
Irf4	*Tcfec*	*Nfic*	*Hmga2*	*Smad1*
Prdm1	*Pou2af1*	*Jun*	*Pgr*	*Tff3*
Stat4	*Prdm1*	*Postn*	*Rxra*	*Pax3*
Xbp1	*Ikzf1*	*Nfe2l1*	*Hoxa5*	*Lef1*
Hmga2	*Cebpg*	*Nfkb1*	*Ar*	*Ctf1*
Atf3	*Postn*	*Irf2*	*Dmrt1*	*Cebpd*
Ets2	*Irf4*	*Usf1*	*Zfp148*	*Zeb1*
Rel	*Aire*	*Srebf1*	*Casr*	*Hmga2*
Hmga1	*Foxp1*	*Arnt*	*Nkx2-1*	*Cebpg*
Rfx1	*Rbpj*	*Nfe2l2*	*Fosb*	*Cebpb*
Sfpi1	*Irf8*	*Nfe2*	*Etv4*	*Atf3*
Myb	*Junb*	*Ahr*	*Nr3c1*	*Elk4*
Rfxank	*Pou2f2*	*Itgal*	*Ikzf1*	*Nfatc3*
Aire	*Pou6f1*	*Fosb*	*Atf3*	*Zbtb7b*
Nfatc2	*Rel*	*Hif1a*	*Yy1*	*Nfatc1*
Stat3	*Yy1*	*Sp2*	*Gr*	*Sox4*
Gfi1	*Srf*	*Max*	*Jund*	*Cux1*
Cebpe	*Stat1*	*Pou2f3*	*Rfx1*	*Cebpa*
Ikzf1	*Irf2*	*Foxo1*	*Sp2*	*Mxd1*
Gata3	*Hp*	*Pou1f1*	*Myb*	*Max*
Traf4	*Mtf1*	*Sp3*	*Stat5b*	*Tcf3*
Pou2f3	*Foxp3*	*En1*	*Hmga1*	*Hmga1*
Fli1	*Gata1*	*Sp4*	*Mybl2*	*Pax8*
Ddit3	*Stat5a*	*Runx2*	*Stat5a*	*Pdx1*
Bach2	*Pax5*	*Gli2*	*Vdr*	*Foxd1*

Nfkb1	*Runx3*	*Sp1*	*Zeb1*	*Ttf1*
Elk4	*Rest*	*Creb1*	*Hp*	*Nfic*
Gabpa	*Jund*	*Egr1*	*Dbp*	*Hoxa4*
Hp	*Zbtb16*	*Myc*	*Rest*	*Myc*
Etv4	*Crem*	*Foxh1*	*Pitx2*	*Usf2*
Hlf	*Cebpa*		*Ctf1*	*Nfya*
Cux1	*Ppard*		*Ppard*	*Elk3*
Alx1	*Atf3*		*Cebpd*	*Zfp354c*
Zbtb16	*Stat5b*		*Hoxa*	*Mycn*
Mtf1	*Mafb*		*Rara*	*Gcm1*
Nf1	*Ikzf2*		*Bcl6*	*Etv4*
Foxa3	*Fosb*		*Elk1*	*Hoxa*
Pou2f2	*Gata2*		*Ets1*	*Mxi1*
Rest	*Traf4*		*Pparg*	*Runx3*
Elk1	*Sp2*		*Foxf1a*	*E2f5*
Ap3b1	*Itgal*		*Hoxa4*	*Mxd4*
Pou2f1	*Nfe2*		*Junb*	*E2f4*
Maf	*Fos*		*Hoxa3*	*Itgal*
Erg	*Zbtb7b*		*Foxm1*	*Rara*
Nfatc1	*Stat2*		*Tcfap2a*	*Sfn*
Foxf2	*Foxa2*		*Nfyb*	*Pou6f1*
Qrsl1	*Nkx2-1*		*Sox9*	*Nkx3-1*
Foxm1	*Nfil3*		*Gata2*	*Gtf2i*
Tff3	*Fosl2*		*Hes1*	*Foxj1*
Tcf7	*Onecut2*		*Ttf1*	*Nfatc2*
Sf1	*Xbp1*		*Aire*	*Erf*
Mycn	*Tcf7*		*Ppara*	*Cdx2*
Deaf1	*Lef1*		*Nfatc1*	*Myb*
Smad7	*Irf7*		*Lef1*	*Foxc1*
Spz1	*Nfia*		*Gfi1*	*Tcfap2a*
Foxa1	*Max*		*Erf*	*Atf1*
Foxf1a	*Myog*		*Nfatc3*	*Pitx2*
Jund	*Sfpi1*		*Foxa2*	*Nfe2l3*
Foxp3	*Smad7*		*Tcf7l2*	*Hoxa5*
Bcl6	*Mxd1*		*Max*	*Foxa1*
Usf1	*Pparg*		*Hoxa7*	*Onecut2*
Mef2d	*Smad1*		*Nr1h2*	*Mxd3*
Atf1	*Stat3*		*Hnf4a*	*Zbtb16*
Stat5a	*Kitl*		*Sp4*	*Mybl2*
Sp2	*Hes1*		*Tcf4*	*Gfi1*
Stat5b	*Gata6*		*Wt1*	*Rfxank*
Mpp1	*Creb1*		*Gata3*	*Sox10*
Crem	*En1*		*Onecut1*	*Tef*

Stat6	*Ets2*	*Foxj1*	*Onecut1*
Nrf1	*Ets1*	*Qrsl1*	*Tcf7*
Cebpg	*Cebpb*	*Foxh1*	*Ovol2*
Mitf	*Jun*	*Pax5*	*Fosb*
Hltf	*Elk4*	*Stat3*	*Atf4*
Ets1	*Pxn*	*Ap3b1*	*Foxa2*
Cebpd	*Gli2*	*Sp3*	*Fosl2*
Pr	*Sox2*	*Foxo4*	*Rest*
Mxd3	*Wt1*	*Cebpa*	*Hoxa7*
Hoxa9	*Hnf1a*	*Foxo1*	*Nfyb*
Tcfec	*Elf1*	*E2f3*	*Gr*
Max	*Nf1*	*Irf1*	*E2f3*
Mzf1	*Smad3*	*Egr1*	*Nfe2*
Pou1f1	*Rara*	*Maf*	*Pgr*
Pdlim5	*Rfx5*	*Cebpb*	*Ppard*
Zeb1	*Pax8*	*Runx2*	*Maf*
Pou3f1	*Ppara*	*Creb1*	*Hp*
Gata2	*Pde6b*	*Cebpg*	*Gli2*
Srf	*Sfn*	*Fos*	*Zfp628*
Hsf1	*Mxd3*	*Otx2*	*Gcm2*
Nr5a2	*Gata4*	*Hoxa9*	*Jund*
Gata1	*Vdr*	*Trp53*	*Sox6*
Itgal	*Hif1a*	*Irf9*	*Junb*
Irf6	*Tbp*	*Otx1*	*Nfyc*
Hoxa4	*Myc*	*Tal1*	*E2f2*
Tgif1	*Ar*	*Gli2*	*Irf7*
Trp53	*Trp53*	*Srf*	*Esr1*
Bach1	*Pou2f1*	*Nfkb1*	*Crx*
Cebpa	*Esr1*	*Sfpi1*	*Xbp1*
Gr	*Runx1*	*Gata4*	*Sp2*
Nfil3	*Egr1*	*Sp1*	*Qrsl1*
Sfn	*Mafk*	*Kitl*	*Mafg*
Foxa2	*Egr2*	*Jun*	*Irf5*
Hoxa7	*Qrsl1*	*Srebf1*	*Foxa3*
Cebpb	*Pou5f1*	*En1*	*Ahr*
Sp3	*Myod1*	*Gli3*	*Nfkb2*
Mxd1	*Sp1*	*Brca1*	*Casr*
Hnf1b	*Runx2*	*Myc*	*Gc*
E2f4	*Smad2*	*Sox10*	*Desp2*
Hoxa	*Casr*	*Ahr*	*Erg*
Myc	*Ahr*	*Myf6*	*Egr1*
Pax5	*Ctf1*	*Stat1*	*Maz*
Hnf1a	*Srebf1*	*Myog*	*E2f1*

Nr3c1	*E2f1*	*Stat2*	*Irf8*
Junb	*Rxra*	*Sox2*	*Ap3b1*
E2f5	*Nr3c1*	*Stat4*	*Irf1*
Tfdp1	*Cdx2*	*Pax6*	*Foxp3*
Ttf1	*Elk1*	*Pr*	*Stra13*
Crx	*Nr1i2*	*Hnf1b*	*Dmrt1*
Mafb	*Cbfb*	*Foxn1*	*Atf2*
Rara	*Sp4*	*Tbp*	*Ikzf2*
Zbtb7b	*Foxo3*	*Nf1*	*Gfi1b*
Postn	*Myb*	*Pxn*	*Gabpa*
Sp1	*Hltf*	*Stat6*	*Tcf7l2*
Hoxa5	*Ebf2*	*Tal2*	*Yy1*
Fosb	*Tead1*	*Gata1*	*Pou2f2*
Tcfap2a	*Hmga2*	*Foxo3*	*Irf3*
Runx1	*Sf1*	*Itgal*	*Fosl1*
Foxh1	*Foxh1*	*Hnf1a*	*Arnt*
Glis1	*Pax4*		*Rora*
Dmrt1	*Atf2*		*Srf*
Cdc37	*Etv4*		*Egr3*
Gata6	*Gr*		*Tlx2*
Dbp	*Erg*		*Rarg*
Smad4	*Pou2f3*		*Trp53*
Pou5f1	*Hmga1*		*Mafk*
Runx3	*Ap3b1*		*Etv6*
E2f2	*Cp2*		*Sox3*
Pparg			*Stat1*
Pou4f1			*Nr1h3*
Rbpj			*Ar*
Rarg			*Rxra*
Gata4			*Sox9*
Gc			*Nfe2l1*
Fos			*Lhx3*
Myod1			*Elk1*
Foxo3			*Hes1*
Sox2			*Mtf1*
Smad3			*Arnt2*
Vdr			*Jun*
En1			*Zfhx3*
Jun			*Foxm1*
Ppara			*Sp3*
Pxn			*Gata1*
E2f3			*Rxrb*
Kitl			*Gata2*

Ppard	*Nfkb1*
Ahr	*Nr5a1*
Smad2	*Pou1f1*
Msx1	*Hnf1b*
Tbp	*Tcfe3*
Brca1	*Ep300*
Hes1	*Bcl6*
Ar	*Fos*
Creb1	*Nkx2-1*
Egr2	*Cbfb*
Hif1a	*Nr2f1*
Esr1	*Fli1*
Nkx2-1	*Stat3*
Smad5	*Nanog*
E2f1	*Tbx5*
Nhlh1	*Dbp*
Runx2	*Pxn*
Nfe2l2	*Pcbp1*
Tpx2	*Rel*
Egr1	*Elf1*
Foxd1	*Sp1*
Rxrb	*Stat4*
Pgr	*Hoxa9*
Srebf1	*Gata3*
Myf5	*Mef2a*
Hnf4a	*Runx1*
Nr1i2	*Stat5a*
Pax6	*Creb1*
Gli2	*Zic1*
Rxra	*Wt1*
Casr	*Hif1a*
Pde6b	*Pou3f3*
Cdx2	*Irf4*
Arnt	*Srebf1*
Gli3	*Gli1*
Hoxa3	*Stat6*
Smad9	*Hnf1a*
Pax2	*Tcf4*
Wt1	*Ppara*
Arnt2	*Hsf1*
Ebf2	*Vdr*
Rax	*Nr1i3*
Ctf1	*Pax2*

Runx2
Gata6
Nf1
Mef2d
Tal1
Pou5f1
Hnf4a
Mitf
Pou3f1
Sf1
Nr1i2
Brca1
Nfe2l2
Tcfeb
Myf5
Tead1
Usf1
Kitl
Nr2f2
Stat5b
Gata4
Pax6
Gli3
Sox2
Egr2
Sfpi1
Sry
Pr
Myog
Mef2c
Gata5
Nkx2-5
Pax5
Foxo3
Tbp
Ebf2
En1
Myf6
Meis1
Pbx1
Ets2
Hlf
Otx2

	Ascl1
	Myod1
	Ets1
	Foxo1
	Hic1
